# Detecting mpox infection in the early epidemic: an epidemiologic investigation of the third and fourth cases in Korea

**DOI:** 10.4178/epih.e2023040

**Published:** 2023-03-23

**Authors:** Taeyoung Kim, Eonjoo Park, Jun Suk Eun, Eun-young Lee, Ji Won Mun, Yunsang Choi, Shinyoung Lee, Hansol Yeom, Eunkyoung Kim, Jongmu Kim, Jihyun Choi, Jinho Ha, Sookkyung Park

**Affiliations:** 1 Capital Regional Center for Disease Control and Prevention, Korea Disease Control and Prevention Agency, Seoul, Korea; 2 Disease Management Division, Deogyang-gu Public Health Center, Goyang, Korea; 3 Seoul National University Bundang Hospital, Seongnam, Korea

**Keywords:** Monkeypox, Contact tracing, Needlestick injuries

## Abstract

**OBJECTIVES:**

As few mpox cases have been reported in Korea, we aimed to identify the characteristics of mpox infection by describing our epidemiologic investigation of a woman patient (index patient, the third case in Korea) and a physician who was infected by a needlestick injury (the fourth case).

**METHODS:**

We conducted contact tracing and exposure risk evaluation through interviews with these 2 patients and their physicians and contacts, as well as field investigations at each facility visited by the patients during their symptomatic periods. We then classified contacts into 3 levels according to their exposure risk and managed them to minimize further transmission by recommending quarantine and vaccination for post-exposure prophylaxis and monitoring their symptoms.

**RESULTS:**

The index patient had sexual contact with a man foreigner during a trip to Dubai, which was considered the probable route of transmission. In total, 27 healthcare-associated contacts across 7 healthcare facilities and 9 community contacts were identified. These contacts were classified into high (7 contacts), medium (9 contacts), and low (20 contacts) exposure risk groups. One high-risk contact was identified as a secondary patient: a physician who was injured while collecting specimens from the index patient.

**CONCLUSIONS:**

The index patient visited several medical facilities due to progressive symptoms prior to isolation. Although the 2022 mpox epidemic mainly affected young men, especially men who have sex with men, physicians should also consider mpox transmission in the general population for the timely detection of mpox-infected patients.

## INTRODUCTION

Mpox, a viral zoonosis, can spread from human to human through close contact with respiratory secretions, skin lesions of an infected person, or recently contaminated objects [1,2]. Human mpox infection was first identified in 1970 in the Democratic Republic of the Congo, and since then, most cases have been reported from forested parts of Central and West Africa where the mpox virus is endemic. In early May 2022, mpox cases were reported in countries where the disease had not been frequently reported and continued to spread in other non-endemic countries [3]. The outbreak primarily affected young men, especially gay, bisexual, and other men who have sex with men, and transmission through skin and mucosal contact during sexual activities was most commonly reported [4].

On November 14, 2022, a probable mpox case was reported at a secondary hospital in Korea. A 24-year-old woman who had recently traveled to the United Arab Emirates in early November 2022 had a single sexual contact with a man foreigner in Dubai. She did not notice any symptoms on the date of return to Korea. After the return, she visited several healthcare facilities due to progressive symptoms, including fever, chill, pain on urination, vaginal discharge, multiple skin and mucosal lesions, and extreme pain from the lesions. Considering the symptoms and exposure history, polymerase chain reaction (PCR) tests were conducted, based on a suspicion of mpox infection. On November 15, the case was laboratory-confirmed as the third mpox case in Korea. While collecting specimens from the patient’s skin lesions, a physician sustained a needlestick injury to her index finger. Three days after exposure, a single skin lesion was formed at the site of the injury, which was confirmed positive on an mpox PCR test.

This report describes (1) the processes of contact tracing and exposure risk evaluation for the case of the woman patient, who did not belong to any group known to be at high risk for mpox (such as men who have sex with men), and (2) the features of the case of the healthcare worker infected through occupational exposure. We thus aimed to inform healthcare professionals about the characteristics of recent mpox cases.

## MATERIALS AND METHODS

### Contact tracing and exposure investigation

In order to collect clinical and epidemiologic information about the index patient, we conducted interviews and contact tracing. We held an initial interview with the index patient to obtain baseline information, including her family and business status and exposure history. To obtain further information, especially related to sexual contact, we designated an interviewer to communicate alone with the index patient.

The Epidemic Investigation Support System in Korea, which was originally established for coronavirus disease 2019 (COVID-19) contact tracing, and then expanded in June 2022 to include mpox and other infectious diseases, enabled us to obtain cellphone-based global positioning system (GPS) records and credit card transaction records from related public authorities and private agents and medical facility and pharmacy visit records from the Drug Utilization Review system of the National Health Insurance Review and Assessment Service [5,6]. We analyzed the GPS and transaction records to cross-check the target places to be investigated with the interview results. We also analyzed medical visit records to estimate the infectious period by identifying the onset of symptoms and their progression. To identify probable contacts and evaluate their risk of exposure, we reviewed closed-circuit television (CCTV) records of the target places and interviewed their personnel.

As healthcare facilities visited by the index patient during the symptomatic period were identified, we visited the facilities and requested the patient’s medical records and facilities’ CCTV records, the latter to assess for possible contacts and related factors, such as contact type and duration. To obtain more accurate information, we interviewed healthcare workers who cared for the index patient, during which we asked about the patient’s symptoms at the time of visit, treatment and procedures given to the patient, their medical opinions, and possible occurrences of contact in the examination rooms.

Anyone who had contact with the index patient during the symptomatic period in any location other than a healthcare facility was considered a community contact. Utilizing the patient’s statement and GPS and credit card records, all locations visited by the patient were identified, including restaurants, pharmacies, and other stores. Field investigations and reviews of CCTV records were conducted at these locations in order to evaluate their exposure risk. Probable household and workplace contacts were also interviewed for the evaluation of their exposure risk.

### Exposure risk evaluation

Risk assessment and classification of contacts were based on the national mpox response guideline (Table 1) [7]. Considering the mode and duration of exposure and other factors, we categorized contacts into high-risk, medium-risk, and low-risk groups. In the case of healthcare workers, we also observed the appropriateness of their personal protective equipment, distances kept from the patient when providing care, and types of procedures conducted. We also reviewed whether there were any exposed visitors whose visiting times overlapped with that of the index patient.

### Monitoring and management of contacts

Contacts who were classified into the high-risk group were recommended to be vaccinated for post-exposure prophylaxis within 4 days from exposure. Those within 5 days to 14 days of exposure and those in the medium-risk group within 4 days of exposure were allowed to be vaccinated. Those who met the criteria for receiving prophylaxis were provided with related information, and vaccinations were given only to volunteers.

Contacts classified into the high-risk or medium-risk group were monitored for 21 days from their last exposure. We recommended self-quarantine for the high-risk group and monitored all high-risk and medium-risk contacts twice a day via phone calls for any new symptoms related to mpox.

According to the guideline, those in the low-risk are guided to self-report when any new symptoms develop during the 21 days following their last exposure. However, we aimed to enhance the monitoring as we lacked experience with this condition and were dealing with exposures in healthcare facilities, which carried elevated potential risks. Accordingly, for contacts in this low-risk group, we checked their symptoms on the last day of their monitoring period.

### Ethics statement

This study was approved by the Institutional Review Board of Korea Disease Control and Prevention Agency (2023-01-01-P-A) and was conducted in accordance with the ethical standards set forth in the Declaration of Helsinki 1964 and its later amendments.

## RESULTS

### Investigation of the index case

In the initial investigation, the index patient reported no significant close contact with confirmed mpox patients or animals, but did report a single sexual contact with a man foreigner during her trip. In additional face-to-face and phone interviews, she stated her sexual orientation as heterosexual, and denied any other sexual contact in the past 3 months, with the exception of condomless intercourse with the partner in Dubai. She did not notice any symptoms in the partner.

After her symptoms developed, she communicated with the partner via a social network service to check his status; he denied any symptom development or known sexually transmitted infections. Partner notification was recommended during the interviews and was conducted. However, we had limitations in identifying his exact status as he lived abroad.

Before the patient’s isolation, she visited 6 clinics and 2 hospitals via the emergency rooms, including a dental clinic (Table 2). On November 8, she visited an emergency room due to chills, dizziness, and dyspnea, which were the first symptoms she noticed, and supportive care was provided. On the same day, she received a regular checkup for her braces at a dental clinic.

The following clinic visits were for the purpose of managing different symptoms. On the third day of her illness, she visited a gynecology clinic for dysuria and was prescribed empirical antibiotics to treat cystitis. Since her systemic condition persisted, she visited another clinic on the same day and was provided with supportive treatment. The next day, she visited a proctology clinic due to experiencing constipation, anal bleeding, and pain. The proctologist noticed her anogenital skin lesions during the examination, and referred her to another gynecology clinic. Empirical antiviral treatment was prescribed at both clinics.

**Figure 1.**
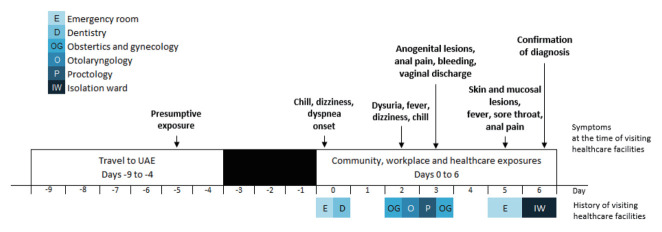
The index patient’s symptoms progress on visiting healthcare facilities. UAE, United Arab Emirates.

On November 13, she visited a second hospital via the emergency room for aggravated symptoms. Multiple skin and mucosal lesions occurred on the face, tongue, trunk, and anogenital area, and systemic symptoms and pain in the lesion areas were exacerbated. Conventional sexually transmitted infections were excluded by gynecologists during the emergency room visit due to the atypical characteristics of the anogenital lesions. The next day, dermatologists reviewed her record prior to her visit and reported her as a possible case of mpox infection. Specimens from her lesions, pharynx, and blood were collected after isolation and confirmed positive on mpox PCR tests (Figure 1). After 11 days of isolation and 9 days of treatment with tecovirimat, her discharge was confirmed by an infectious disease physician, based on the status of the lesions, negative PCR test results, and relief of symptoms.

### Contact tracing

Based on interview results, the index patient’s household and workplace contacts were identified. She lived with 6 family members, including her parents and 4 siblings, and shared common spaces, including toilets, a living room, and a kitchen. They shared towels and dishes during her symptomatic period, which was classified as a high-risk environmental contact. One of her siblings shared a bedroom with her, and the mother accompanied her on the hospital visits. We estimated that there were enough close contacts between her and her family members for potential viral transmission via droplets, fomites, and direct contact with the patient’s lesions. Dozens of coworkers at the small company where she worked shared an office and toilet, and 3 of them had desks close to the patient’s desk.

It was confirmed that the patient had visited 7 healthcare facilities and 13 other locations during her symptomatic period, including restaurants, pharmacies, and other stores. All healthcare facility workers identified as contacts were evaluated for their risk of infection. The possibility of transmissions in each facility varied by procedure type, appropriateness of personal protective equipment, and duration of potential exposures. Dental, anal, and pelvic examinations during the visits were considered probable risks, and contact with her urine specimen was also considered at risk.

After the index patient was isolated, all healthcare workers who cared for the patient were equipped with appropriate protective equipment, including face shields or goggles, gowns, masks, and gloves. Yet, there was a significant exposure resulting from an accident: one of the patient’s doctors was injured by a needlestick during specimen collection on November 14.

Community contacts from visits to the other (non-healthcare facility) locations were also identified. Cashiers, pharmacists, and customers who visited stores at the same time were deemed at minimal risk of transmission due to the face mask mandate that was in place in Korea at the time [8]. No direct physical contact was revealed in any of these visits.

### Exposure risk evaluation of individual contacts

Based on the exposure risk evaluations of the index patient’s contacts, a total of 36 contacts were classified into contact groups to be managed: 7 high-risk, 9 medium-risk, and 20 low-risk contacts (Table 3).

All 6 household contacts were categorized as belonging to the high-risk group, based on their intimate household settings and behaviors. The other high-risk contact was the 33-year-old woman physician who sustained a needlestick injury during collection of the patient’s skin lesion specimens.

A total of 8 healthcare workers and 1 patient were exposed to a medium-level risk of infection in varying settings, including the possibility of transmission via droplets, fomites, and direct contacts, while 17 healthcare workers were classified as low-risk contacts as they used proper protective equipment. Three of the patient’s coworkers were classified into the low-risk group, based on the low possibility of direct contact in the workspace.

### Monitoring and management of contacts

Among all identified contacts, 2 high-risk and 2 medium-risk contacts experienced symptoms during their monitoring period. The needlestick-injured contact experienced mild systemic symptoms and a lesion at the injury site 3 days after the accident. An initial PCR test showed a negative result, but mpox infection was confirmed by a follow-up test on November 22. The index patient’s mother had non-specific symptoms, including sore throat, headache, and dizziness, but had a negative result on her mpox PCR test. One medium-risk contact had diarrhea and fever, and the other had a skin rash and an itching sensation; they were not suspected of having mpox infections, given the non-specificity of these symptoms.

All 7 high-risk contacts were recommended to be vaccinated for post-exposure prophylaxis according to their exposure histories. The physician was vaccinated 20 hours after the injury with a single dose of a third-generation smallpox vaccine (single-dose subcutaneous injection of JYNNEOS 0.5 mL, manufactured by Bavarian Nordic Tuborg Havn, Denmark). The index patient’s mother considered vaccination due to her symptoms, but declined after receiving a confirmed negative result on her mpox PCR test. The other family members refused to be vaccinated.

### Contact tracing for the secondary case (the needlestick-injured physician)

After the exposure, the needlestick-injured patient was treated with tecovirimat for 14 days and isolated for 26 days, after which she was discharged based on her systemic and lesion statuses [9]. During the interview following her test confirmation, she denied any form of close contact or casual community contact aside from the injury. According to her statement, she noticed her injury immediately, and thus tried to minimize any potential transmission risks by wearing a face mask, putting a bandage on the injury site, and limiting person-to-person contacts. Contact tracing confirmed that she visited her house, the hospital where she worked, a convenience store, and a restaurant after exposure. No household or community contacts were identified. Healthcare workers in the hospital were revealed as having no significant chance of transmission as their contact durations were within 5 minutes, face masks were worn, and no physical contact was made. Therefore, none of the secondary patient’s hospital coworkers was classified into the high-risk, medium-risk, or low-risk contact group for monitoring.

## DISCUSSION

The index patient with recent travel to the United Arab Emirates had a single sexual contact with a man foreigner in Dubai. Upon her return, she visited several healthcare facilities due to progressive symptoms, including fever and multiple skin and mucosal lesions. Contact tracing and exposure risk evaluation were conducted: 36 contacts across 7 healthcare facilities and community settings were identified, including 7 high-risk, 9 medium-risk, and 20 low-risk contacts. One high-risk contact who was needlestick-injured was vaccinated for post-exposure prophylaxis within 20 hours after exposure and confirmed positive for mpox 8 days after the injury. Among all other contacts, 1 high-risk and 2 medium-risk contacts experienced symptoms during their monitoring period, yet none developed mpox.

No other secondary cases were identified apart from the needlestick-injured patient; however, there were some possibilities of transmission that were not fully evaluated. First, the exact source of infection was not determined. We could not estimate the possibility of transmission during her trip, since there was no report of mpox infection in the United Arab Emirates after the 16 cases reported through July 2022 [10]. However, considering that another confirmed mpox case was reported after travel to Dubai in October 2022, transmission during the index patient’s trip remains a possibility [11]. Despite the sexual contact in Dubai being the most probable mode of transmission, it is possible that the partner may not have been the infector since he gave negative responses to questions on symptoms or known sexually transmitted infections. To clarify his status, it would have been ideal for overseas interviews or international investigations to have accompanied our investigation. Furthermore, if he were confirmed to not be the infector, the target period of investigation would then be extended to 21 days before symptom onset to identify any additional chances of transmission, including sexual contacts, invasive procedures, or other risk factors.

It took 6 days from symptom onset to diagnose the index patient, which suggests that measures to reduce chances of transmission should be hereafter reinforced. First, mpox infection was not initially considered as a diagnosis because no cases were reported in Dubai for several months, as mentioned above. Five of the 8 healthcare facilities visited by the index patient during her symptomatic period took note of her recent travel history in the International Traveler Information System. It provides information on country visit histories for 6 infectious diseases, including Middle East respiratory syndrome (MERS), COVID-19, Lassa fever, plague, Ebola virus disease, and mpox, to healthcare personnel at medical institutions and pharmacies [12,13]. Outbreak countries are regularly redesignated, and messages are provided according to the diseases. Since the United Arab Emirates was only classified as a MERS and not an mpox outbreak country in November 2022, the only precautions provided to physicians were notifications regarding possible MERS cases.

There were also difficulties in diagnosing the index patient’s mpox infection in its early phase since she did not notice skin and mucosal lesions at the onset of her systemic symptoms. It was less likely that the physicians would suspect an mpox infection at that moment as a thorough evaluation of transmission risk, including taking exposure history, may not be routinely conducted for differential diagnosis of non-specific symptoms such as chills and dizziness.

Furthermore, the clinical characteristics of mpox infection were not well-known to most physicians practicing in Korea, since only 2 cases had been reported prior to the index patient’s case. When her anogenital lesions were observed at clinics, evaluations for common sexually transmitted infections were conducted and empirical antivirals were prescribed, rather than considering the possibility of an mpox infection. In addition, the fact that the index patient visited 6 different medical facilities to try to relieve her symptoms before the eventual diagnosis may have hindered the differential diagnosis of her illness since physicians could not observe her symptom progression due to fragmented medical services without follow-up.

Lastly, woman patients tend not to be suspected of mpox infection without identification of significant contacts with mpox-confirmed patients since the 2022 mpox epidemic in non-endemic countries has mainly affected man patients, especially men who have sex with men. However, mpox infection in woman patients has also been reported continuously around the world, and some of them were suspected of having occurred via routes other than sexual contact, such as household or non-sexual close contacts or occupational exposures, according to a global case series [14]. In that study, one-third of woman mpox patients were initially misdiagnosed. Considering this result, our case report suggests that physicians should consider mpox infection when diagnosing the general population, especially, but not exclusively, woman patients who are sexually active or those with equivalent risk factors, including invasion of intact skin or mucosa. In addition, healthcare personnel should be aware of the possibility of transmission via occupational exposure to mpox virus and implement appropriate safety practices for managing sharps, given the case reports of transmission by needlestick injuries during the 2022 outbreak of mpox [15-18].

Thus, our report suggests that physicians should be well-informed of clinical and epidemiological characteristics of mpox for rapid detection. Evaluation of distinguishable lesions and taking thorough histories of potential exposures may aid physicians in diagnosing mpox patients in the early phase. Related information should be properly communicated to the public and physicians.

## Figures and Tables

**Table 1.** Response measures by exposure risk level

Exposure risk level	Definition	Measure	Post-exposure prophylaxis
High	Unprotected direct contact or high-risk environmental contact	21 day of active monitoring (check status twice a day via text message or phone call)	Recommended (within 4 day after exposure)
		Self-quarantine recommended	Allowed (between 5 to 14 day after exposure)
Medium	Unprotected exposure to infectious materials or droplets, or potential exposure to aerosols	21 day of active monitoring	Allowed (within 4 day after exposure)
		Quarantine not required	
Low	Protected physical or droplet exposure	Monitoring not required	Not applicable
	No physical contact and little chance of exposure to droplets	Quarantine not required	
		Inform of precautions	

**Table 2.** The index patient's medical visit history during the symptomatic period

Visit date	Type of healthcare facility	Symptoms and observed status	Differential diagnosis
Nov 8	Hospital (emergency room A)	Chill, dizziness, dyspnea	-
Nov 8	Clinic (dentistry)	Regular checkup (braces)	-
Nov 10	Clinic (obstetrics and gynecology A)	Dysuria	Cystitis
Nov 10	Clinic (otolaryngology)	Fever, chill, dizziness	COVID-19
Nov 11	Clinic (proctology)	Anal pain, anal bleeding, constipation	Herpes simplex virus infection
			Varicella zoster virus infection
Nov 11	Clinic (obstetrics and gynecology B)	Anogenital lesions, vaginal discharge, dysuria, anal pain, night sweat	Herpes simplex virus infection
Nov 13	Hospital (emergency room B)	Skin and mucosal lesions (face, tongue, chin, chest, back, shoulder, labium major, anus), fever, sore throat, anal pain	Mpox

**Table 3.** Results of exposure risk evaluation of the index patient's contacts according to risk level

Location		Contact type (n)	Situation	Exposure duration	Personal protective equipment	
					Mask	Gloves
High-risk						
	Hospital (isolation ward)	Doctor (1)	Needlestick injury	-	○	○
	Household	Family member (6)	Shared home and everyday items such as dishes and towels	11 day	Ⅹ	Ⅹ
Medium-risk						
	Dental clinic	Dental staff (2)	Dental examination	5-24 min	○	○
	Gynecology clinic A	Doctor (1)	Examination with inappropriate mask-wearing	5 min	△	Ⅹ
		Nurse (1)	Injection, contact with urine specimen without gloves	10 min	○	Ⅹ
	Proctology clinic	Doctor (1)	Anal examination	15 min	○	○
	Gynecology clinic B	Doctor (1)	Pelvic examination using colposcope and forceps	10 min	○	○
		Nurse (1)	Exam assistant, disinfection of colposcope and forceps	10 min	○	○
		Nurse (1)	Direct contact with skin during injection	3 min	○	Ⅹ
		Patient (1)	Use of the same exam table before disinfection (1-min duration)	-	-	-
Low-risk						
	Workplace	Coworker (3)	No direct contact and little conversation	4 day	○	Ⅹ
	Emergency room A	Healthcare worker (6)	Vital sign check, blood collection, ECG application, insertion and removal of venous line	5-10 min	○	Ⅹ
	Otolaryngology clinic	Doctor (1)	Examination	15 min	○	○
		Nurse (2)	Exam assistant	15 min	○	Ⅹ
	Proctology clinic	Nurse (1)	Exam assistant	15 min	○	Ⅹ
	Emergency room B	Healthcare worker (7)	Vital sign check, blood collection, insertion and removal of venous line, nasopharyngeal swab	5-20 min	○	○

○, applied appropriately; △, applied inappropriately; Ⅹ, not applied; ECG, electrocardiogram.
